# Survival after hospital discharge in patients hospitalized for acute coronavirus disease 2019: data on 2586 patients from a tertiary center registry

**DOI:** 10.3325/cmj.2022.63.335

**Published:** 2022-08

**Authors:** Marko Lucijanić, Nevenka Piskač Živković, Marko Zelenika, Mislav Barišić-Jaman, Ivana Jurin, Ana Matijaca, Nikola Zagorec, Marko Lagančić, Besa Osmani, Iva Bušić, Sara Šakota, Ivan Vukoja, Ivica Lukšić, Bruno Baršić

**Affiliations:** 1 Hematology Department, Dubrava University Hospital, Zagreb, Croatia; 2 Primary Respiratory and Intensive Care Center, Dubrava University Hospital, Zagreb, Croatia; 3 University of Zagreb, School of Medicine, Zagreb, Croatia; 4 Pulmonology Department, Dubrava University Hospital, Zagreb, Croatia; 5 Department of Gastroenterology, Hepatology and Clinical Nutrition, Dubrava University Hospital, Zagreb, Croatia; 6 Cardiology Department, Dubrava University Hospital, Zagreb, Croatia; 7 Endocrinology Department, Dubrava University Hospital, Zagreb, Croatia; 8 Nephrology Department, Dubrava University Hospital, Zagreb, Croatia; 9 Department of Emergency and Intensive Care Medicine, Dubrava University Hospital, Zagreb, Croatia; 10 Gastroenterology and Nephrology Department, General County Hospital Požega, Požega, Croatia; 11 Faculty of Medicine Osijek, Josip Juraj Strossmayer University of Osijek, Osijek, Croatia; 12 Department of Maxillofacial Surgery, Dubrava University Hospital, Zagreb, Croatia

## Abstract

**Aim:**

To assess the long-term survival after hospital discharge of patients hospitalized due to coronavirus disease 2019 (COVID-19).

**Methods:**

We retrospectively reviewed data on post-discharge survival of 2586 COVID-19 patients hospitalized in our tertiary hospital from March 2020 to March 2021.

**Results:**

Among 2586 patients, 1446 (55.9%) were men. The median age was 70 years, interquartile range (IQR, 60-80). The median Charlson comorbidity index was 4 points, IQR (2-5). The median length of hospital stay was 10 days, IQR (7-16). During a median follow-up of 4 months, 192 (7.4%) patients died. The median survival time after hospital discharge was not reached, and 3-month, 6-month, and 12-month survival rates were 93%, 92%, and 91%, respectively. In a multivariate analysis, mutually independent predictors of worse mortality after hospital discharge were age >75 years, Eastern Cooperative Oncology Group status 4, white blood cell count >7 ×10^9^/L, red cell distribution width >14%, urea on admission >10.5 mmol/L, mechanical ventilation during hospital stay, readmission after discharge, absence of obesity, presence of chronic obstructive pulmonary disease, dementia, and metastatic malignancy (*P* < 0.05 for all).

**Conclusion:**

Substantial risk of death persists after hospital admission due to COVID-19. Factors related to an increased risk are older age, higher functional impairment, need for mechanical ventilation during hospital admission, parameters indicating more pronounced inflammation, impaired renal function, and particular comorbidities. Interventions aimed at improving patients’ functional capacity may be needed.

Coronavirus disease 2019 (COVID-19) affects multiple organ systems, but its clinical presentation is dominated by respiratory symptoms and high rates of respiratory deterioration (1). The disease strains health care systems and affects both hospital and ambulatory care. Up to 15%-20% of pre-vaccination patients were hospitalized due to respiratory insufficiency or active comorbidities that required hospital care (2). Many people still remain unvaccinated, presenting a high risk group for short-term mortality (3). Elderly patients and those burdened with comorbidities are especially susceptible to unfavorable clinical course during hospital stay (4). Data on survival subsequent to hospital discharge after overcoming respiratory insufficiency are scarce. The main concerns affecting these patients are death, rehospitalizations, and reduced functioning (5,6). Due to the importance of the period after hospital discharge for patients’ rehabilitation and social re-inclusion, health care providers and caregivers need to be informed on the risks associated with this period. Thus, we aimed to assess the mortality and risk factors for unfavorable outcome after hospital discharge among COVID-19 patients treated in our institution.

## Patients and methods

The presented data are part of the Registry project encompassing clinical and laboratory data as well as the outcomes of all hospitalized COVID-19 patients treated in Dubrava University Hospital during the COVID-19 pandemic. Dubrava University Hospital was repurposed into a regional tertiary COVID-19 center. Of 4102 hospitalizations and 4014 consecutive COVID-19 patients treated from March 2020 to March 2021, 2586 patients were discharged and are included in the analysis. All patients were whites. All had positive polymerase chain reaction (PCR) or antigen COVID-19 test before hospital admission. During hospital stay, patients were treated according to the contemporary guidelines with various exposure to low molecular weight heparin, corticosteroids, and remdesivir. Post-discharge follow-up and survival were assessed through a review of patients’ electronic records of post-discharge follow-up visits and through telephone check-up. COVID-19 disease severity on admission was graded based on the World Health Organization (WHO) recommendations. The study was approved by the Institutional Review Board of Dubrava University Hospital (2021/2503-04).

### Statistical analysis

The normality of distribution of numerical variables was tested with the Kolmogorov-Smirnov test. Numerical variables are presented as median and interquartile range. Categorical variables are presented as frequencies and percentages. The receiver operating characteristic (ROC) curve analysis was used to define optimal cut-off levels for numerical variables regarding survival prediction. The presented survival curves and estimates are based on the Kaplan-Meier method. Univariate and multivariate survival associations were assessed with the Cox regression analysis. Variables were selected with the backward approach, with *P* < 0.05 and *P* > 0.1 inclusion and exclusion criteria, respectively. *P* values <0.05 were considered statistically significant. All analyses were performed with the MedCalc statistical software, version 20.006 (MedCalc Software Ltd, Ostend, Belgium).

## Results

### Patients' characteristics

We enrolled 2586 patients who were discharged from hospital after index admission due to COVID-19. The median age was 70 years, IQR (60-80). A total of 1446 (55.9%) patients were men. The median Charlson comorbidity index was 4, IQR (2-5). Severe or critical COVID-19 symptoms on admission were present in 1956 (75.6%) patients. The median time from the start of COVID-19 symptoms to admission was 5 days, IQR (1-10), and the median length of hospital admission was 10 days, IQR (7-16).

During the median follow-up of 4 months, 192 (7.4%) patients died. The median survival time after hospital discharge was not reached. Three-month, 6-month, and 12-month survival rates were 93%, 92%, and 91%, respectively.

### Associations of demographic parameters and medical history with post-discharge survival

**Figure 1:**
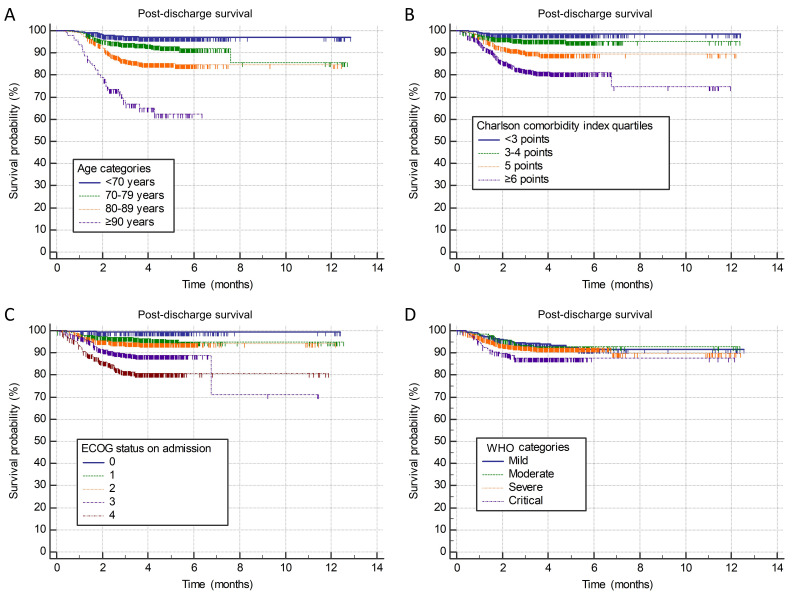
Post-discharge survival stratified according to the **A)** age categories (patients <70 years of age and ≥90 years of age were grouped together due to similar outcomes in younger patients and small number of patients/events in the elderly), **B)** Charlson comorbidity index quartiles, **C)** Eastern cooperative oncology group (ECOG) status on admission, and **D)** World Health Organization (WHO) disease severity categories.

Higher mortality after hospital discharge was significantly associated with older age (hazard ratio [HR] 1.07, *P* < 0.001) (Figure 1A). According to the ROC curve analysis, the optimal cut-off for survival discrimination was >75 years, with older patients having a higher risk of post-discharge mortality (HR 4.51, 95% confidence interval [CI] 3.3-6.14; *P* < 0.001). Sex was not significantly associated with post-discharge survival (*P* = 0.166).

Charlson comorbidity index was significantly associated with higher post-discharge mortality (HR 1.34; *P* < 0.001). The ROC curve analysis yielded the cut-off level of >4 points, above which the patients experienced a higher risk of death (HR 5.29, 95% CI 3.79-7.37; *P* < 0.001) (Figure 1B). The frequency of individual comorbidities and their relationship with post-discharge survival are shown in Table 1.

**Table 1:** Demographic and medical history parameters and their relationship with post-discharge survival (N = 2586)*

		Univariate association with survival	
	**n (%)^†^**	**HR and 95% CI**	P
**Age** (years); median (IQR)	70 (60-80)	1.07 (1.06-1.09)	<0.001
**Male sex**	1446 (55.9)	0.81 (0.62-1.09)	0.166
**Charlson comorbidity index**; median (IQR)	4 (2-5)	1.34 (1.28-1.41)	<0.001
**Arterial hypertension**	1707 (66)	1.41 (1.03-1.95)	0.032
**Diabetes mellitus**	727 (28.1)	0.97 (0.7-1.33)	0.833
**Obesity**	708 (27.4)	0.51 (0.35-0.75)	<0.001
**Chronic renal disease**	256 (9.9)	1.89 (1.29-2.77)	0.001
**COPD**	164 (6.3)	1.83 (1.16-2.92)	0.010
**Congestive heart failure**	318 (12.3)	2.16 (1.54-3.03)	<0.001
**Atrial fibrillation**	357 (13.8)	2.85 (2.09-3.89)	<0.001
**Liver cirrhosis**	20 (0.8)	2.22 (0.71-6.9)	0.172
**Dementia**	357 (13.8)	4.32 (3.23-5.77)	<0.001
**Active malignant disease**	233 (9)	2.37 (1.64-3.42)	<0.001
**Prior or active malignancy**	412 (15.9)	1.74 (1.25-2.42)	0.001
**Metastatic malignant disease**	141 (5.5)	2.92 (1.93-4.42)	<0.001
**Autoimmune/rheumatic disease**	109 (4.2)	0.23 (0.06-0.92)	0.038
**History of VTE**	99 (3.8)	0.79 (0.35-1.78)	0.565
**History of CVI**	253 (9.8)	2.61 (1.84-3.7)	<0.001
**History of MI**	199 (7.7)	0.88 (0.5-1.54)	0.654
**History of bleeding**	127 (4.9)	1.43 (0.81-2.51)	0.212
**VTE during hospitalization**	155 (6)	0.96 (0.51-1.82)	0.904
**MI during hospitalization**	28 (1.1)	1.0 (0.25-4.08)	0.996
**CVI during hospitalization**	57 (2.2)	2.6 (1.37-4.92)	0.003
**GI tract bleeding during hosp.**	66 (2.6)	2.08 (1.06-4.06)	0.032

*Abbreviations: HR – hazard ratio, CI – confidence interval, COPD – chronic obstructive pulmonary disease, VTE – venous thromboembolism, CVI – cerebrovascular infarction, MI – myocardial infarction, GI – gastrointestinal.

†if not otherwise indicated.

### Relationship of COVID-19 disease severity, clinical status on admission, and hospital stay parameters with post-discharge survival

The indication for hospital admission and the referral origin were significantly associated with post-discharge mortality. Patients with asymptomatic COVID-19 (admitted due to non-medical and social reasons, HR 2.2; P 0.008), patients admitted due to a neurologic indication (HR 2.08; *P* = 0.004), and nursing-home residents (HR 3.95; *P* < 0.001) experienced a worse post-discharge survival.

A worse post-discharge survival was significantly associated with a shorter disease duration on admission (HR 0.94; *P* < 0.001), worse Eastern Cooperative Oncology Group (ECOG) status on admission (HR 1.88; *P* < 0.001), higher symptom severity on presentation (HR 1.2; *P* = 0.046), other infection on admission (HR 2.09; *P* < 0.001), higher white blood cell count (WBC; HR 1.03; *P* < 0.001), lower hemoglobin (HR 0.98; *P* < 0.001), higher red blood cell distribution width (RDW; HR 1.13; *P* < 0.001), lower estimated glomerular filtration rate (eGFR; HR 0.98; *P* < 0.001), higher urea (HR 1.04; *P* < 0.001), and higher D-dimer on admission (HR 1.28; *P* < 0.001). Post-discharge survival stratified according to the admission ECOG status and WHO disease severity categories are shown in Figure 1C and 1D, respectively.

Hospital stay length negatively affected post-discharge prognosis (HR 1.03; *P* < 0.001), and patients requiring >11 days of hospital stay experienced higher post-discharge mortality (HR 2.05, 95% CI 1.54-2.73; *P* < 0.001). Mechanical ventilation survivors (HR 3.72; *P* < 0.001) and patients who experienced prolonged immobilization during hospital stay (≥7 days without bathroom privileges HR 2.56; *P* < 0.001) had a worse post-discharge survival. Higher mortality was also observed in patients requiring hospital readmission (HR 4.92; *P* < 0.001). Pneumonia, need for oxygen therapy, high-flow oxygen therapy, or intensive care unit stay were not significantly associated with post-discharge survival (Table 2).

**Table 2:** Parameters related to coronavirus disease 2019 hospital admission and their relationship with post-discharge survival (N = 2568)*

		Univariate association with survival	
	n (%)	HR and 95% CI	P
**Indication for admission**			
asymptomatic	85 (3.3)	2.2 (1.24-3.93)	0.008
pneumonia	1610 (62.3)	Reference category	
temperature without pneumonia	95 (3.7)	0.73 (0.29-1.79)	0.489
acute medical condition	356 (13.8)	0.33 (0.81-1.86)	0.327
acute neurological condition	136 (5.3)	2.08 (1.27-3.44)	0.004
acute surgical condition	304 (11.8)	0.98 (0.61-1.59)	0.964
**Origin of referral**			
Patient’s home	1018 (39.4)	Reference category	
Nursing home	223 (8.6)	3.95 (2.54-6.15)	<0.001
Other hospital	1345 (52)	2.03 (1.43-2.88)	<0.001
**Disease duration on admission** (days)	5 (1-10)	0.94 (0.91-0.97)	<0.001
**ECOG status on admission**			
0	307 (11.9)	Reference category	
1	659 (25.5)	6.06 (1.43-25.6)	0.014
2	648 (25.1)	8.55 (2.05-35.6)	0.003
3	545 (21.1)	17.8 (4.36-73.35)	<0.001
4	427 (16.5)	31.6 (7.75-128.76)	<0.001
**MEWS score**	2 (1-3)	1.09 (1.01-1.19)	0.036
**WHO severity on admission**			
mild	433 (16.7)	Reference category	
moderate	197 (7.6)	1.04 (0.54-2.01)	0.914
severe	1744 (67.4)	1.19 (0.79-1.82)	0.393
critical	212 (8.2)	1.99 (1.16-3.43)	0.013
**Admission WBC** (× 10^9^/L)	7.4 (5.5-10.3)	1.03 (1.02-1.05)	<0.001
**Admission hemoglobin** (g/L)	129.5 (115-142)	0.98 (0.97-0.99)	<0.001
**Admission RDW** (%)	13.8 (13.2-14.9)	1.13 (1.09-1.18)	<0.001
**Admission platelets** (× 10^9^/L)	230 (172-305)	0.99 (0.99-1.0)	0.169
**Admission CRP**	72.2 (27.8-131.9)	1.0 (0.99-1.0)	0.239
**Admission ferritin**	640 (347-1132)	0.99 (0.99-1.0)	0.648
**Admission eGFR**	79.2 (55.4-94.2)	0.98 (0.97-0.99)	<0.001
**Admission urea** (mmol/L)	7.1 (5.2-10.3)	1.04 (1.03-1.05)	<0.001
**Admission D-dimers** (mg/L FEU)	1.14 (0.63-2.62)	1.28 (1.14-1.43)	<0.001
**Pneumonia**	2124 (82.1)	1.37 (0.91-2.06)	0.131
**Other infection on admission**	324 (12.5)	2.09 (1.49-2.95)	<0.001
**Oxygen therapy**	1872 (72.4)	1.3 (0.93-1.82)	0.124
**High flow oxygen therapy**	192 (7.4)	1.15 (0.68-1.95)	0.598
**Mechanical ventilation**	74 (2.9)	3.72 (2.23-6.22)	<0.001
**Need for ICU**	240 (9.3)	1.21 (0.76-1.93)	0.409
**Length of hospitalization**	10 (7-16)	1.03 (1.02-1.04)	<0.001
**Immobilization ≥7 days**	739 (28.6)	2.56 (1.93-3.41)	<0.001
**Readmission to the hospital**	76 (2.9)	4.92 (3.16-7.68)	<0.001

*Abbreviations: HR – hazard ratio, CI – confidence interval, ECOG – Eastern cooperative oncology group, MEWS – modified early warning score, WHO – World health organization, WBC – white blood cell count, RDW – red blood cell distribution width, CRP – C reactive protein, eGFR – estimated glomerular filtration rate, ICU – intensive care unit.

### Multivariate analysis of factors affecting post-discharge survival

The variables significantly univariately associated with post-discharge mortality (excluding Charlson comorbidity index and including specific comorbidities) were included in the Cox regression model building process by using the backward approach. Numerical variables were dichotomized at optimal cut-off levels determined by using the ROC curve analysis (Table 3). Mutually independent predictors of a worse post hospital discharge mortality were age >75 years, ECOG status 4, WBC>7 ×10^9^/L, RDW>14%, urea >10.5 mmol/L on admission, mechanical ventilation during hospital stay, readmission post discharge, absence of obesity, presence of chronic obstructive pulmonary disease, dementia, and metastatic malignancy.

**Table 3:** Cox regression analysis model investigating mutually independent associations of patient-related and hospital stay-related parameters with survival after hospital discharge

	Multivariate association with survival	P
Variable	HR and 95% CI	
**Age**>75 years	2.92 (1.9-4.48)	**<0.001**
**ECOG status** 4	1.94 (1.3-2.9)	**0.001**
**WBC**>7 ×10^9^/L	1.5 (1.02-2.21)	**0.039**
**RDW**>14%	1.89 (1.26-2.84)	**0.002**
**Urea**>10.5 mmol/L	1.81 (1.24-2.64)	**0.002**
**Mechanical ventilation**	8.68 (4.71-15.97)	**<0.001**
**Readmission post discharge**	5.28 (2.92-9.54)	**<0.001**
**Obesity**	0.47 (0.29-0.78)	**0.003**
**Chronic obstructive pulmonary disease**	2.06 (1.15-3.72)	**0.016**
**Dementia**	2.19 (1.45-3.32)	**<0.001**
**Metastatic malignancy**	3.23 (1.89-5.55)	**<0.001**
**History of cardiovascular insult**	1.58 (0.99-2.51)	0.055

*Abbreviations: HR – hazard ratio, CI – confidence interval, ECOG – Eastern cooperative oncology group, WBC – white blood cell count, RDW – red blood cell distribution width.

## Discussion

Our study showed that hospital treatment for COVID-19 led to prolonged health risks. In the post-discharge period, patients experienced substantial mortality, especially those with critical presentation of disease on admission and older patients with specific comorbidities. Similar long-term sequelae have been reported in other viral illnesses (7).

In our study, patients admitted to hospital from nursing homes and referred from other institutions had worse post-discharge outcomes than patients coming from home and those admitted due to acute neurological conditions and non-medical/social reasons (thus marked as asymptomatic) rather than due to COVID-19 pneumonia *per se*. Patients referred from other institutions and those coming from nursing homes were significantly older and more burdened with comorbidities, as well as had a significantly worse functional status on admission, than patients coming from home, which is probably the reason for our finding since these patients had less severe COVID-19 symptoms on admission (data not shown). No association with CRP on admission was found despite the association with other measures of increased inflammation, such as higher D-dimer and RDW. Nevertheless, a composite CRP-based index determined on admission has been shown to predict long-term outcomes (8). As the current study shows, the intensity of COVID-19 symptoms quantified with the modified early warning score at hospital admission and mechanical ventilation requirement have negative prognostic value in the post-discharge period. Systemic inflammatory response associated with severe acute respiratory syndrome coronavirus 2 infection might lead to a deterioration in the existing comorbidities (9) after the recovery of respiratory self-sufficiency.

Lower hemoglobin levels and higher RDW, which are recognized negative prognostic factors for acute COVID-19 infection (10,11), were also shown to affect the outcomes in the post-discharge period. A similar role was observed for parameters of renal excretion function. The quality of hematopoiesis and parameters of renal excretion function, as universal markers of overall health fitness, were shown to be prognostic in a number of chronic and malignant diseases in the pre-COVID era (12-14). Higher urea may indicate worse hydration status on admission, especially considering that it outperformed the presence of chronic kidney disease and creatinine in a multivariate prognostic model. Leukocytosis, as a non-specific parameter of physical stress, also seems to be associated with a worse long-term prognosis. Nevertheless, platelets, which might play an important role in the protection of lung parenchyma during infection (15), were not associated with post-discharge survival.

Sex, which significantly affects the survival of patients in the hospital setting (4), was not associated with post-discharge survival, which is contrary to other reports (16). However, older age and higher comorbidity burden evidently affected the post-discharge outcomes. Post-discharge mortality could be considered as a surrogate measure of cardiovascular events, especially considering that COVID-19 might increase long-term cardiovascular risks (17). In the current study, particularly detrimental effects on post-discharge survival were evident in patients with dementia, cardiovascular, pulmonary, and malignant diseases. These comorbidities highly impair daily activities and functional independence. Indeed, the measures of functional status on admission (like ECOG scale), as well as during hospital stay (immobilization without bathroom privileges due to imposed or objective medical reasons) and the need for a prolonged hospital stay all negatively affected post-discharge survival. These findings agree with other reports that recognize impaired functional status as an important negative consequence of COVID-19 infection after hospital treatment for acute disease (5,6,16,18). Functional status is a potentially modifiable factor, and preventive measures and targeted interventions aimed at its improvement might contribute to better outcomes of patients with a higher mortality risk. The need for rehospitalization also predicted a worse post-discharge survival, which highlighted the fact that patients with long-term mortality risk are frail and might need home health services or chronic care (16).

Limitations of our study are single-center experience, retrospective study design, and the fact that it was conducted in a pandemic period characterized by low vaccination rates. The data were collected in a high-volume tertiary center treating the most severe cases of COVID-19 and patients who required urgent medical care for other conditions but also tested positive for SARS-CoV-2. The patients treated in our institution might not be directly comparable to those from other cohorts due to ethnical and regional differences, and due to differences in patient profile. Our results should be primarily interpreted as representative of Croatian population. Furthermore, the follow-up period was relatively short. Despite these limitations, our results provide valuable and unique insights into the post-discharge period risks in patients previously hospitalized for COVID-19.

In conclusion, the hospital stay for COVID-19 leads to a substantial risk of death post discharge. Patients with increased risk for death are those of older age, higher functional impairment, need for mechanical ventilation, parameters indicative of more pronounced inflammation, impaired renal function, and particular comorbidities. These patients might require interventions aimed at improvement of functional capacity.
